# Incidental Coronary Artery Calcification and the Risk of Major Adverse Cardiovascular Outcomes

**DOI:** 10.7759/cureus.73531

**Published:** 2024-11-12

**Authors:** Daniel Antwi-Amoabeng, Bryce D Beutler, Munadel Awad, Moutaz Taha, Kashmala Syed, Sri Harsha Boppana, Joban Ghuman, Jasmine Ghuman, Sunil Sathappan, Mitch Pisane, Mark B Ulanja, Vijay Neelam, Nageshwara Gullapalli, Chanwit Roongsritong, Omar Canaday

**Affiliations:** 1 Internal Medicine, Christus Ochsner St. Patrick Hospital, Lake Charles, USA; 2 Radiology, University of Southern California Keck School of Medicine, Los Angeles, USA; 3 Internal Medicine, Renown Regional Hospital, Reno, USA; 4 Internal Medicine, University of Nevada Reno School of Medicine, Reno, USA; 5 Internal Medicine, Veterans Affairs (VA) Sierra Nevada Health Care System, Reno, USA; 6 Rheumatology, University of Nevada Reno School of Medicine, Reno, USA; 7 Hematology/Oncology, Cleveland Clinic, Cleveland, USA; 8 Cardiology, University of Utah, Salt Lake City, USA; 9 Cardiology, Renown Institute for Heart and Vascular Health, Reno, USA

**Keywords:** chest ct, computed tomography, coronary artery calcification, coronary artery calcium, major adverse cardiovascular events, mortality

## Abstract

Background

Incidental findings of coronary artery calcifications (CACs) are not consistently reported, and the clinical significance relating to cardiovascular outcomes remains to be established. In this single-center cross-sectional study, we assessed the association between incidental coronary artery calcification documented on formal chest CT reports and the incidence of major adverse cardiovascular events (MACE).

Methods

A MACE was defined as the occurrence of stroke or transient ischemic attack or ST-segment elevation myocardial infarction, non-ST-elevation myocardial infarction, or undergoing coronary artery bypass grafting. A composite endpoint included either MACE or the occurrence of cardiovascular death. We assessed the predictors of the composite outcome and the effect of lipid-lowering therapy on the composite outcome in the studied cohort.

Results

The composite outcome occurred in 39.1% of the 1,354 subjects studied. Peripheral arterial disease was the only comorbid condition associated with increased odds (adjusted odds ratio (aOR) 2.6, p < 0.001, 95% CI: 1.9 - 3.56). The average treatment effect of lipid-lowering therapy was 0.11 (p = 0.002, 95% CI: 0.04 - 0.17). At 10 years after the first CAC report, the presence of peripheral artery disease appears to present the lowest odds of survival, which is <50% (hazard ratio (HR) 2.44, p < 0.001, 95% CI: 1.67 - 3.56).

Conclusion

In patients with CAC on incidental chest CT scans, the presence of peripheral arterial disease is associated with increased odds of MACE and/or cardiovascular death. In those with incidental CAC on non-gated chest CT scans, the residual risk for MACE remains high despite lipid-lowering therapy and antiplatelet agents.

## Introduction

Electrocardiogram (ECG)-gated coronary artery calcification (CAC) by Agatston score is the gold standard for quantification of radiologically apparent coronary calcifications [1]. Results from this formal study are used to enhance risk stratification by traditional risk calculators such as the atherosclerotic cardiovascular disease (ASCVD) and Framingham Risk Score [2]. Indeed, the Multi-Ethnic Study of Atherosclerosis (MESA) risk score calculator is based on the combination of CAX and traditional risk factors [3]. These scores have predictive value for future major adverse cardiovascular events (MACE) and guide discussion about the initiation of primary prevention pharmacotherapy and other risk factor management strategies such as targeted lifestyle modification in asymptomatic adults [4,5]. Although the popularity of dedicated computed tomography (CT) studies of CACs seems to be increasing, as evidenced by recommendations for its adoption by the major cardiovascular societies, there are far more routine non-gated CT studies of the chest in the general population [6,7]. The chest is among the top two targets of CT acquisition in the primary care/emergency department population, and the annual acquisition of routine, non-gated chest CTs outnumbers dedicated coronary artery calcium studies by a factor of 15 [8,9]. Dedicated coronary calcium studies have two drawbacks. First, there is the added out-of-pocket expense, as most insurance companies do not pay for this test, which can range from US$ 100 to 400 [10]. Second, a dedicated study exposes patients to excess radiation dose, which can be variable depending on study protocol and carries an estimated lifetime radiation-related excess cancer risk of 42 cases per 100,000 men and 62 cases per 100,000 women with a median dose of 2.3 mSv [11].

In addition to chest CT images acquired in the emergency department and primary care populations, data from low-dose CT for pulmonary nodule surveillance provide a unique opportunity to increase the utility of non-contrast, non-gated chest CTs for identifying patients at risk for MACE. The images provide good visualization of the coronary vessels in addition to the primary indication of nodule surveillance. The sensitivity of non-gated CT for CAC is similar to those of dedicated gated studies [12]. However, radiologists are inconsistent in reporting incidental CACs on routine chest CT studies [13]. Thus, clinicians may be missing an opportunity to intervene in the progression of atherosclerotic cardiovascular disease. To answer the question, “Should clinicians be concerned about incidental coronary artery calcification on a chest CT?" we assessed the association between radiologically apparent coronary calcification on non-gated chest CTs and the occurrence of MACE and/or cardiovascular cause of death in this cross-sectional study. We also assessed the time to outcome of interest and assessed the influence of lipid-lowering therapy on the outcomes.

## Materials and methods

Study subjects

**Figure 1:**
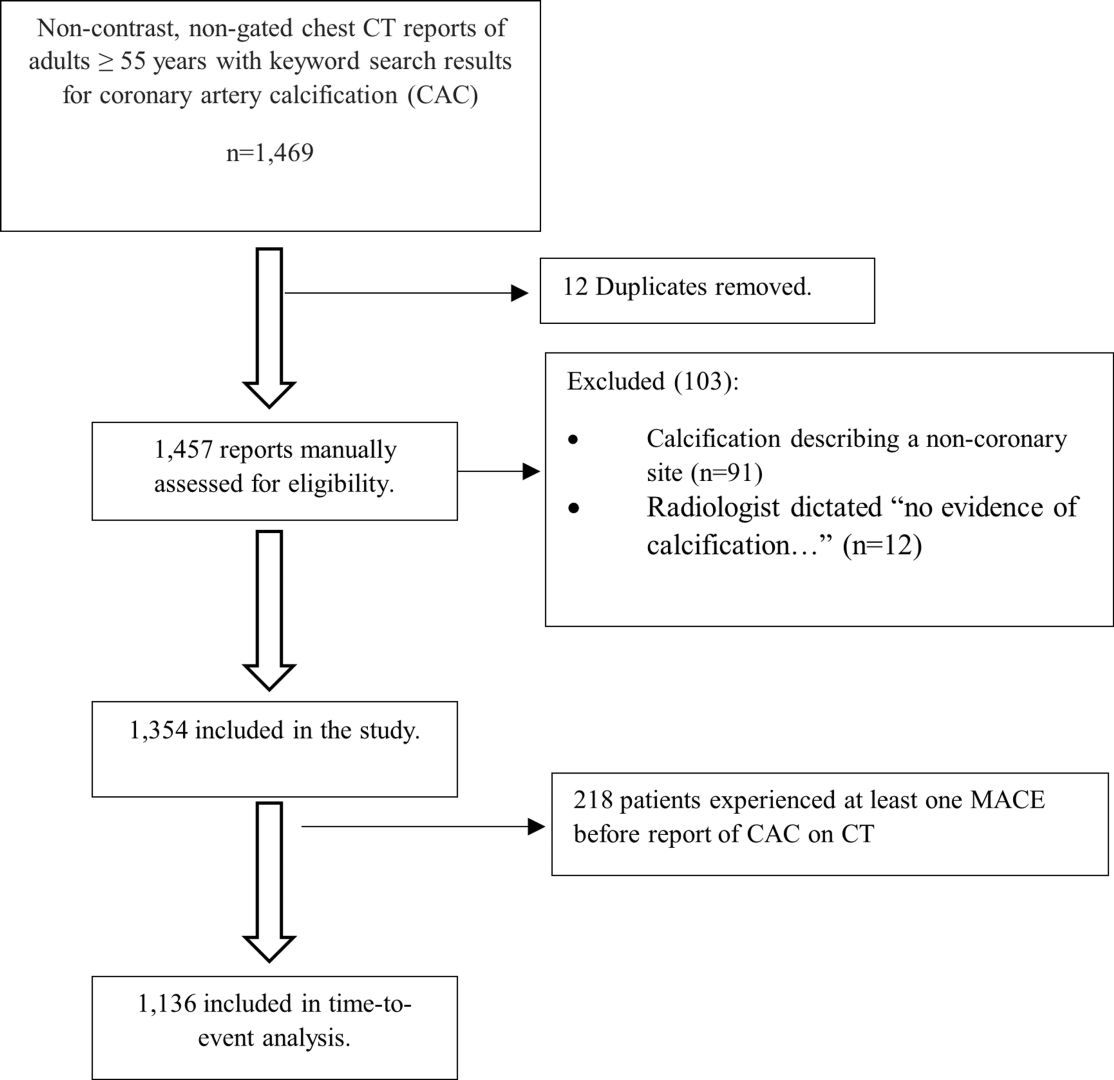
Flow diagram of subject selection and inclusion in the study CAC: coronary artery calcification; MACE: major adverse cardiovascular events; CT: computed tomography

Subjects were selected by a Boolean syntax-aided search of the non-contrast, non-gated chest CT report database at Renown Regional Medical Center, a 946-bed tertiary care hospital in Reno, Northern Nevada. Keyword search terms were “coronary artery calcification”, “coronary atherosclerosis”, “calcified coronary artery/arteries.” We restricted the search period to January 2009 through December 2019 and included subjects at least 55 years old at the time of the first image acquisition. A total of 1,469 records were retrieved after the initial search. After removing 12 duplicates, the remaining 1,457 records were manually assessed for eligibility using keyword-enhanced scanning of individual reports. We excluded 103 subjects whose reports either described a non-coronary calcification site or had a dictated phrase “no evidence of calcification..." The remaining 1,354 patients were included in the study. Of these, 218 experienced at least one MACE prior to the index report of CAC and were not included in a subsequent time-to-event analysis. Figure 1 summarizes the patient selection process. Subject data such as sex, age, chronic comorbid conditions, and chronic prescription medication list were abstracted from a systematic review of the medical records. Comorbid conditions and medications of interest have been previously described [14-16]. Outcomes of interest included MACE, which was defined as the occurrence of stroke or transient ischemic attack or ST-segment elevation myocardial infarction, non-ST-segment elevation myocardial infarction, or undergoing coronary artery bypass graft. A composite endpoint included either MACE or the occurrence of cardiovascular death. The endpoint was adjudicated by carefully reviewing all medical records, including primary care clinician notes, emergency room encounter notes, admission history and physical notes, and discharge or death summaries. In time-to-event analysis, we modeled the influence of comorbidities and medications on the composite outcome.

Statistical analyses

Baseline patient characteristics were summarized as mean ± standard deviation, median (interquartile range (IQR)) as appropriate, or as proportions. Differences in the observations were assessed using the Student's t-test or Chi-square test of proportions as appropriate. We used the Chi-square goodness-of-fit test to assess whether the observed proportions differ from the equi-probability model (that MACE types should occur at equal frequencies). The cardiovascular cause of death was determined by the discharging physician’s documentation of a cardiovascular etiology as the primary or secondary cause of death in the death summary. We assessed the influence of patient demographic features, comorbid conditions, and chronic outpatient medications on the composite endpoint.

The effect of lipid-lowering therapy (use of at least one of the listed medications) on the composite endpoint was estimated using regression adjustment estimators of treatment effect in the entire sample and those who received lipid-lowering therapy. Regression adjustment accounts for the non-random assignment of treatment when estimating the effect of a treatment using observational data. In time-to-event analyses, we performed a Cox proportional hazards regression to model the effect of comorbid conditions and chronic medication use on the hazard for composite outcome. For covariate selection, we used the log-rank test of equality across strata for categorical variables and a univariate Cox proportional hazard regression for continuous variables, where appropriate. We tested for the validity of the proportional hazard assumption with appropriate post-estimation operations.

We checked and accounted for the presence of collinearity among the covariates. Initial models included all covariates with significant p-values. The final model was confirmed to be parsimonious and properly fit based on linktest and Akaike’s information criterion post-estimation operations, respectively. All analyses were performed at the two-tailed 5% level of significance using Stata version 16.1 (Stata Corporation, College Station, Texas).

## Results

Baseline characteristics

We identified 1,354 subjects who met the inclusion criteria. There were significantly more males than females in the cohort (60% males vs. 40% females, p <0.001). Among the patients who reached the composite endpoint, 340 (64.1%) were male and 190 (35.9%) were female (p = 0.013). The median age of patients when CAC was first reported was 72 years. The median age at which the composite endpoint was met was 76 years. Comorbid conditions commonly associated with MACE were noted, with several being significantly more common in the composite endpoint group. A comprehensive list of prescription medications was reviewed and summarized in Table 1. A total of 855 (63.2%) patients in our cohort had at least one lipid-lowering agent prescribed during the study period. Statins were the most frequently prescribed medication of this drug group, with 62% of subjects having at least one statin prescribed. Forty-two patients (3.1%) were on ezetimibe, 17 (1.3%) were on gemfibrozil, 14 (1.03%) were on fenofibrate, and 4 (0.3%) were on proprotein convertase subtilisin/kexin type 9 (PCSK9) inhibitors. A very small number of patients were on more than one drug. Table 2 compares the characteristics of patients who received lipid-lowering therapy versus those who were not.

**Table 1:** Baseline patient characteristics of subjects with the composite endpoint compared to those without the endpoint; differences in the observations were assessed using the Student’s t-test, Pearson’s Chi-square test of proportions, or Fisher’s exact test as appropriate at a 5% significance level. CAC: coronary artery calcification; ACEi: angiotensin-converting enzyme inhibitors; ARBs: aldosterone receptor blockers; COPD: chronic obstructive pulmonary diseases; HIV: human immunodeficiency virus

Variable	Overall (n = 1,354)	No composite endpoint (n = 824 (60.9%))	Composite endpoint occurred (n = 530 (39.1%))	p-value	Test statistic
Age (mean ± SD), years					t-test
At first CAC report	71.5 ± 12.1	71.0 ± 12.1	72.3 ± 12.1	0.06	-1.88
At the end of the study/death	75.6 ± 12.1	75.2 ± 12.2	76.3 ± 12.1	0.06	-1.87
Age category	n (%)	n (%)	n (%)		χ^ 2 ^test
Less than 73 years	698 (51.5)	438 (53.2)	260 (49.1)	0.14	2.17
73 years and above	656 (48.5)	386 (46.8)	270 (50.9)		
Sex					
Female	541 (39.7)	351 (42.6)	190 (35.9)	0.01	6.12
Male	813 (60.0)	473 (57.4)	340 (64.1)		
Comorbid conditions					
Atrial fibrillation/flutter	491 (36.3)	268 (32.5)	223 (42.1)	<0.001	12.73
Alcohol use	410 (30.3)	259 (31.4)	151 (28.5)	0.25	1.32
Cancer	411 (30.3)	255 (30.9)	156 (29.4)	0.56	0.35
Chronic renal failure	524 (38.7)	283 (34.3)	241 (45.5)	<0.001	16.83
COPD	523 (38.6)	309 (37.5)	214 (40.4)	0.29	1.13
Diabetes	545 (40.2)	300 (36.4)	245 (46.2)	<0.001	12.93
Heart failure	419 (30.9)	210 (25.5)	209 (39.4)	<0.001	29.37
HIV	9 (0.7)	8 (1.0)	1 (0.2)	0.1	2.99
Hyperlipidemia	778 (57.5)	412 (50)	336 (69.1)	<0.001	47.92
Hypertension	1,071 (79.1)	625 (75.8)	446 (84.1)	<0.001	13.44
Illicit drug use	56 (4.1)	29 (3.5)	27 (5.1)	0.16	2.02
Lupus	12 (0.9)	7 (0.8)	5 (0.9)	1	0.03
Obesity	316 (23.3)	205 (24.9)	111 (20.9)	0.09	2.79
Peripheral arterial disease	238 (17.6)	86 (10.4)	152 (28.7)	<0.001	74.08
Rheumatoid arthritis	47 (3.5)	31 (3.8)	16 (3)	0.47	0.53
Sleep apnea	225 (16.6)	127 (15.4)	98 (18.5)	0.14	2.21
Tobacco use	868 (64.1)	521 (63.2)	347 (65.5)	0.4	0.71
Chronic medications					
ACEi/ARBs	810 (59.8)	443 (53.8)	367 (69.2)	<0.001	32.17
Anticoagulants	504 (37.2)	269 (32.6)	235 (44.3)	<0.001	18.87
Antiplatelets	826 (61.0)	411 (49.9)	415 (78.3)	<0.001	109.54
Beta-blockers	837 (61.8)	434 (52.7)	403 (76.0)	<0.001	74.62
Calcium channel blockers	565 (41.7)	326 (39.6)	239 (45.1)	0.04	4.06
Colchicine	37 (2.7)	20 (2.4)	17 (3.2)	0.39	0.74
Lipid-lowering therapy	855 (63.1)	435 (52.8)	420 (79.2)	<0.001	96.99
Metformin	252 (18.6)	140 (17.0)	112 (21.1)	0.56	3.65
Methotrexate	17 (1.3)	9 (1.1)	8 (1.5)	0.62	0.45
Spironolactone	187 (13.8)	104 (12.6)	83 (15.7)	0.11	2.50

**Table 2:** Comparison of covariates by lipid-lowering therapy (LLT) treatment status; differences in the observations were assessed using Pearson’s Chi-square test of proportions at a 5% significance level. ACEi: angiotensin-converting enzyme inhibitors; ARBs: aldosterone receptor blockers; COPD: chronic obstructive pulmonary diseases; HIV:  human immunodeficiency virus

	No LLT (n = 499 (36.9%))	LLT prescribed (n = 855 (63.1%))	p-value	Test statistic
Variable	n (%)	n (%)		χ^ 2 ^test
Age category			0.15	2.07
Less than 73 years	270 (54.1)	428 (50.1)		
73 years and above	229 (45.9)	427 (49.9)		
Sex				
Female	213 (42.7)	328 (38.4)	0.12	2.45
Male	286 (57.3)	527 (61.6)		
Comorbid conditions				
Atrial fibrillation/flutter	149 (29.9)	342 (40.0)	<0.001	14.02
Alcohol use	166 (33.3)	224 (28.5)	0.07	3.34
Cancer	151 (30.3)	260 (30.4)	0.95	0.0033
Chronic renal failure	145 (29.1)	379 (44.3)	<0.001	30.97
COPD	172 (34.5)	351 (41.)	0.02	5.76
Diabetes	125 (25.1)	420 (49.1)	<0.001	75.93
Heart failure	121 (24.2)	298 (34.8)	<0.001	16.58
HIV	7 (1.4)	2 (0.2)	0.01	6.52
Hyperlipidemia	122 (24.4)	656 (76.7)	<0.001	352.28
Hypertension	328 (65.7)	743 (86.9)	<0.001	85.41
Illicit drug use	26 (5.2)	30 (3.5)	0.13	2.30
Lupus	7 (1.4)	5 (0.6)	0.14	2.40
Obesity	93 (18.7)	223 (26.1)	0.002	9.76
Peripheral arterial disease	52 (10.4)	186 (21.7)	<0.001	27.94
Rheumatoid arthritis	15 (3.0)	32 (3.7)	0.48	0.51
Sleep apnea	58 (11.6)	167 (19.5)	<0.001	14.22
Tobacco use	307 (61.5)	561 (65.6)	0.13	2.29
Chronic medications				
ACEi/ARBs	200 (40.1)	610 (71.3)	<0.001	128.15
Anticoagulants	148 (29.7)	356 (41.6)	<0.001	19.35
Antiplatelets	187 (37.5)	639 (74.7)	<0.001	183.91
Beta-blockers	216 (43.3)	621 (72.6)	<0.001	114.96
Calcium channel blockers	167 (33.5)	398 (46.5)	<0.001	22.18
Colchicine	10 (2.0)	27 (3.2)	0.21	1.58
Metformin	40 (8.0)	212 (24.8)	<0.001	58.57
Methotrexate	5 (1.0)	12 (1.4)	0.52	0.41
Spironolactone	44 (8.8)	143 (16.7)	<0.001	16.55

Outcomes

**Figure 2:**
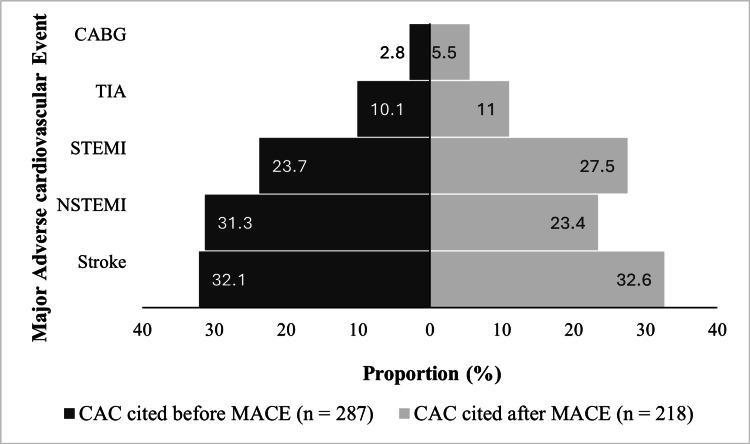
Distribution of MACE type among subjects based on when CAC was first reported on the chest CT CABG: coronary artery bypass graft; CAC: coronary artery calcification; CT: computed tomography; MACE: major adverse cardiovascular event; TIA: transient ischemic attack; STEMI: ST-elevation myocardial infarction; NSTEMI: non-ST-elevation myocardial infarction

**Figure 3:**
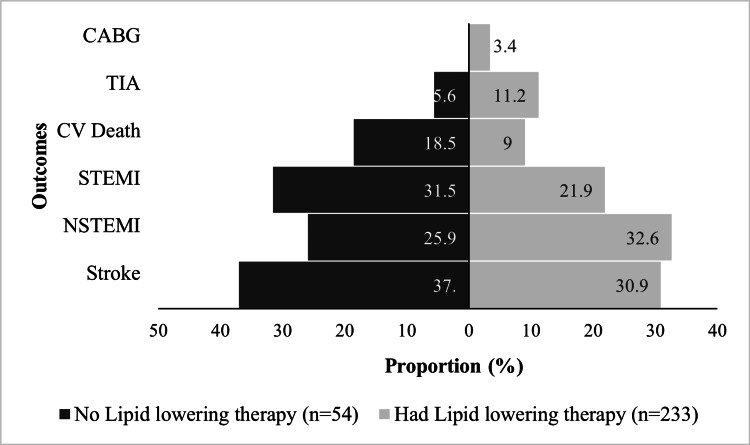
Incidence of outcomes among patients who received lipid lowering therapy compared to those who did not CABG: coronary artery bypass graft; CV: cardiovascular; NSTEMI: non-ST-elevation myocardial infarction; STEMI: ST-elevation myocardial infarction; TIA: transient ischemic attack

A MACE occurred in 505 (37.3%) of the subjects. Of these, 287 had a report of CAC before the MACE. The most common MACE was stroke (32.3%). Coronary artery bypass graft was the least common event (4%). Figure 2 shows the distribution of MACE type according to when CAC was first cited in the CT report. Cardiovascular causes of death occurred in 79 patients, representing 15.2% of those who died. The all-cause mortality rate for our cohort was 8.33%. In most of the cases, 37%, the cause of death was unknown. These subjects may have died outside of the hospital, and/or a cause of death was not documented in the chart. At 20%, infectious etiologies represented the second most common cause of death. Cardiovascular causes of death occurred in 15.2% of the subjects. Cancer represented 8.3%, and hypoxic respiratory failure, non-cardiogenic shock, non-septic shock, and end-stage liver, kidney, and pulmonary diseases as a unit accounted for the remaining 19.5%. The composite endpoint of MACE and/or cardiovascular death occurred in 530 (39.1%) of the subjects, most of whom were male (340 (64.1%)). Event rates of the various types of MACE and death from cardiovascular disease did not differ significantly when comparing those who were on lipid-lowering therapy to those who were not (Figure 3).

Predictors of the composite outcome

**Figure 4:**
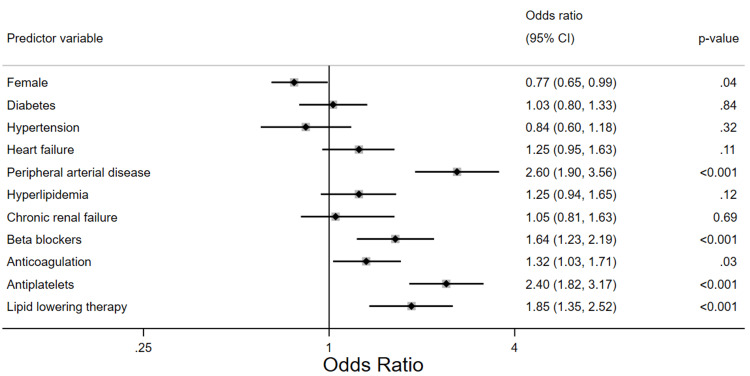
Forest plot showing the adjusted odds of the composite endpoint for all patients included in the study CI: confidence interval

A multivariable logistic regression of the predictors of the composite endpoint demonstrated that the female sex is associated with a 23% reduction in the odds of the composite outcome, adjusted odds ratio (aOR) of 0.77, p-value = 0.04, 95% with a 95% confidence interval of 0.65 - 0.99. Diabetes, hypertension, dyslipidemia, congestive heart failure, chronic renal failure, and atrial fibrillation and/or atrial flutter were not found to have a statistically significant association with the composite endpoint. Peripheral artery disease is associated with more than two-fold greater odds of having the composite outcome (aOR 2.6, p < 0.001, 95% CI: 1.9 - 3.56). Figure 4 shows the adjusted predictive strength of the covariates included in the best-fit model.

Effect of treatment with lipid-lowering therapy

Most of the subjects (855 (63.1%)) in our cohort had at least one lipid-lowering therapy/drug prescribed. Statins were the most frequently prescribed medication of this therapeutic group at 62%. Next to that was ezetimibe with 3.1%, 1.3% were on gemfibrozil, 1% were on fenofibrates, and 0.3% were on PCSK9 inhibitors. In routine practice, lipid-lowering therapy is often implemented in secondary prevention efforts, i.e., after the occurrence of a MACE. Owing to limitations in the data collection, we were unable to determine the temporal relationship between the index prescription of lipid-lowering therapy and the occurrence of CAC on CT and/or MACE. Therefore, to assess the effect of lipid-lowering therapy on the composite endpoint, we employed regression adjustment estimators, which account for the non-random assignment of lipid-lowering therapy in our observational data. Our analysis reveals an average treatment effect (ATE) of 0.11, meaning 11% more of the composite endpoint had the entire population been treated (ATE 0.11, p = 0.002, 95% CI: 0.04 - 0.17). The analysis further showed that had no one been treated (i.e., the potential outcome means), the baseline composite endpoint would have been 0.33 (p <0.001 CI: 0.27 - 0.38). In terms of odd ratios, the ATE is 1.11, p = 0.002, CI: 1.04 - 1.19, and a baseline odds ratio of 1.39, p<0.001, CI: 1.31 - 1.47.

Time-to-event analysis

**Figure 5:**
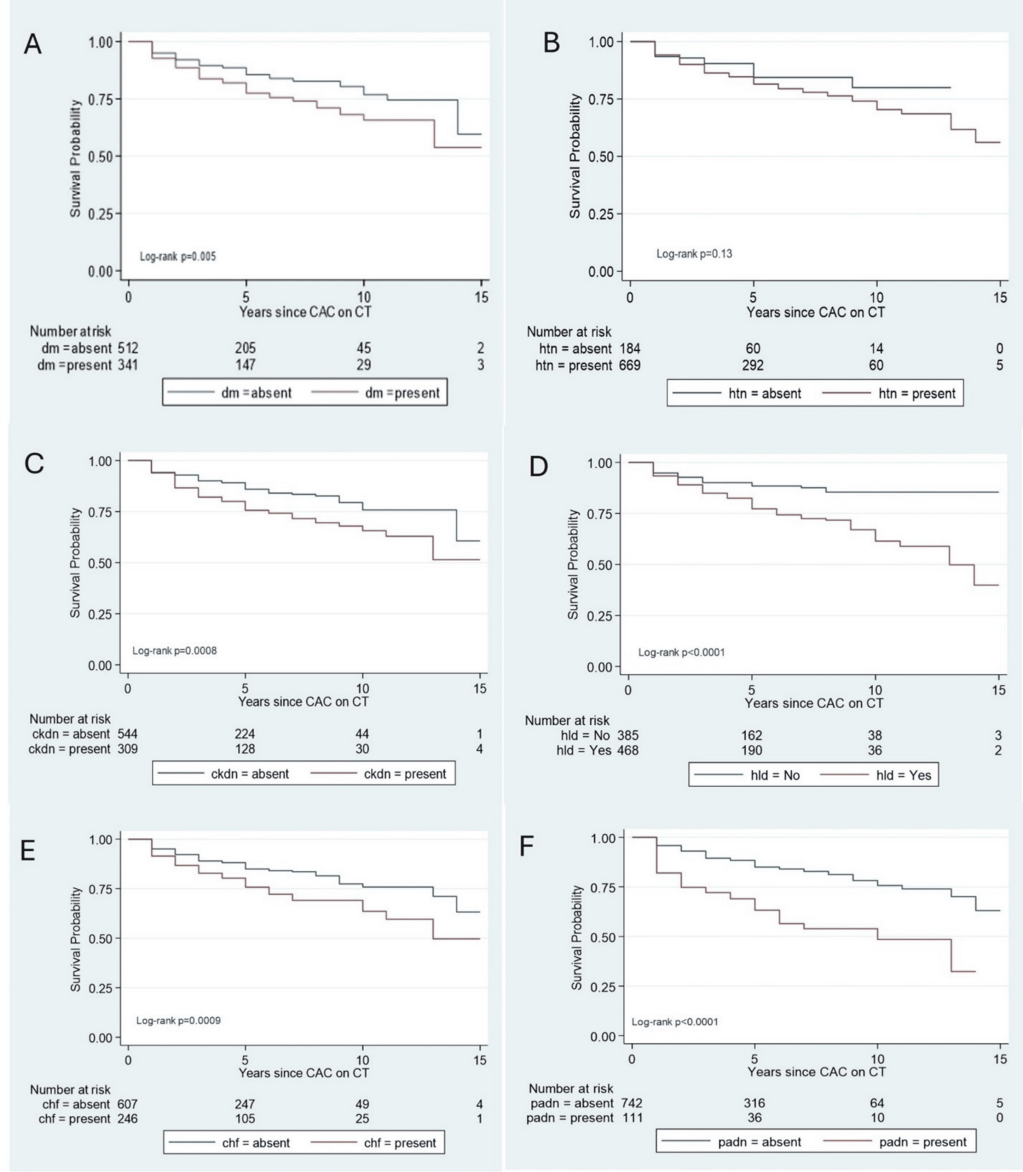
Kaplan-Meier survival plots by patient comorbid conditions Survival rates were compared using the log-rank test at a 5% level of significance. The presence of diabetes (dm) (A), hypertension (htn) (B), chronic kidney disease (ckdn) (C), hyperlipidemia (hld )(D), and congestive heart failure (chf) (E) is associated with a 10-year survival between 50% and 75%. However, the presence of peripheral arterial disease (padn) (F) had a 10-year survival of <50% from the time of initial coronary artery calcification on chest imaging. All comorbid conditions except hypertension were associated with significantly lower survival probabilities.

A total of 1,136 patients were considered for the time-to-event analysis (Figure 1). Of these, 283 had CAC reported during the same hospitalization when the composite endpoint was reached and were excluded from the analysis. Thus, 853 subjects were included in our time-to-event analysis. A total of 144 composite events occurred in 3,932 total analysis times at risk under observation. The median follow-up time was two years, from 0 to 29 years. The mean time-to-event was 4.6 years (median: four years (range: one to 29 years)). Peripheral vascular disease was the only comorbid condition found to be statistically significant (hazard ratio (HR) = 2.44, p-value <0.001, and 95% CI: 1.67 - 3.56). The only chronic medication found to be statistically significant was antiplatelet therapy (HR = 3.18, with a p-value at <0.001 and a 95% CI: 2.01 - 5.05). Table 3 summarizes the result of the model. We constructed Kaplan-Meier survival probability plots for comorbid conditions. Figure 5 shows that all the comorbid conditions except hypertension were associated with significantly lower survival probabilities when present in the cohort. Ten years after the first CAC report, the presence of peripheral artery disease appears to present the lowest odds of survival probability, which is <50%.

**Table 3:** Cox proportional hazard model of the influence of comorbidities and medications on the composite outcome

Variable	Hazard ratio	p-value	95% Confidence intervals
Diabetes	1.15	0.45	0.81 - 1.23
Hypertension	0.62	0.07	0.37 - 1.04
Heart failure	1.15	0.44	0.80 - 1.65
Hyperlipidemia	1.37	0.13	0.91 - 2.05
Chronic renal failure	1.25	0.21	0.88 - 1.78
Peripheral arterial disease	2.44	<0.001	1.67 - 3.56
Illicit drug use	1.94	0.09	0.89 -4.21
Beta-blockers	1.46	0.08	0.96 -2.24
Anticoagulants	1.32	0.11	0.94 - 1.87
Antiplatelets	3.18	<0.001	2.01 - 5.05
Lipid-lowering therapy	1.53	0.07	0.97 - 2.41

## Discussion

Cardiovascular disease is the leading cause of death in the United States and worldwide [17]. Preventive measures, including age-appropriate screening and early initiation of statin therapy, have played a fundamental role in reducing the global burden of disease [18, 19]. In addition, advancements in percutaneous coronary intervention and bypass grafting have improved patient outcomes [20, 21]. Nevertheless, despite remarkable progress, cardiovascular disease remains a major public health concern. Clinical research has traditionally centered on the development of new therapies and surgical and endovascular interventions. However, emerging evidence suggests that leveraging existing technologies, such as the routine chest CT scan, may help identify patients requiring early diagnosis and risk modification, thereby improving cardiovascular risk stratification [22].

The presence of coronary artery calcium on a CT scan of the chest has been established as an important finding that can help guide patient management. The Coronary Artery Risk Development in Young Adults (CARDIA) study was a prospective, community-based study conducted over a three-decade period that aimed to assess the clinical significance of coronary artery calcium among individuals aged 32 to 46 years. The authors found that the presence of any appreciable coronary artery calcium on a CT scan portended a significantly increased risk of coronary heart disease, cardiovascular disease, and death and suggested that the selective use of coronary artery calcium screening could reduce mortality [22]. The findings were corroborated in a subsequent multicenter retrospective cohort study by Miedema et al., which revealed that young adults with coronary artery calcium were at significantly increased risk of coronary heart disease and cardiovascular mortality; the authors concluded that the detection of coronary artery calcium on CT scan may help direct clinical decision-making [23]. Coronary artery calcium has also been shown to be predictive of cardiovascular disease and death among middle-aged adults and the elderly [24-26]. Our study included predominantly elderly patients (median age: 72 years) and added to the previously reported findings pertaining to CAC among the elderly population. However, whereas previous studies included subjects without known cardiovascular disease [26] or used ECG-gated studies and formally calculated CAC using Agatston scores [24, 25], our study included a representative sample of the general population undergoing routine chest CT scans, which is reflective of real-world scenarios frequently encountered in clinical practice.

Over 60 million CT scans are performed annually in the United States, nearly 20% of which image the chest [27]. Indications are broad and include suspected pneumonia, pulmonary embolism, trauma, pericardial disease, and annual lung cancer screening [28]. Dedicated evaluation of the coronary arteries is performed in select patients and involves quantification of calcified and non-calcified coronary artery plaque with risk stratification based on correlation of the degree of coronary artery disease and sociodemographic factors [29]. However, although coronary artery calcium scoring is recommended for primary prevention of cardiovascular disease among borderline and intermediate-risk adults, its use in clinical practice is somewhat limited; among the 10 million chest CT scans performed annually, only 600,000 are dedicated calcium scoring examinations [27,30]. The relative rarity of dedicated calcium scoring examinations is likely related to the distribution of referring providers, as routine CT scans are frequently ordered by general practitioners in a wide range of clinical settings, whereas dedicated calcium scoring scans are typically ordered only by cardiologists for patients with a high likelihood of coronary artery disease or a congenital coronary artery anomaly.

The ECG-gated coronary artery calcium scoring using the Agatston score, the product of coronary artery plaque area and attenuation-weighting factor, represents the gold standard for the assessment of radiologically apparent coronary artery calcium [1]. However, coronary artery calcium can also be evaluated and quantified on routine non-gated, non-contrast-enhanced chest CT scans. Indeed, both the Society of Cardiovascular Computed Tomography (SCCT) and the Society of Thoracic Radiology (STR) recommend that coronary artery calcium be evaluated and reported on all non-contrast chest CT scans [31]. In addition, the American College of Radiology Lung CT Screening Reporting and Data System (Lung-RADS), a standardized reporting system used to describe pulmonary nodules on low-dose CT scan lung cancer screening, includes a designated modifier for incidentally detected coronary artery calcium [32].

The benefits of routine coronary artery calcium assessment on a chest CT scan are two-fold. First, a significantly larger number of patients will undergo a non-contrast chest CT scan using a routine protocol as compared to those undergoing tailored ECG-gated coronary artery calcium scoring, and thus patients without clinical risk factors or classic symptomatology can benefit from early diagnosis and risk modification. Second, the radiation exposure of an ECG-gated coronary artery calcium CT scan is not insignificant, with a median radiation dose of 2.3 mSv; if incidentally detected coronary artery calcium is described on a routine or low-dose chest CT scan, patients can potentially be spared the additional radiation exposure of an ECG-gated coronary artery calcium study [11]. Moreover, coronary artery calcium results described on routine chest CT scans are comparable to those obtained with dedicated ECG-gated coronary artery calcium scoring, and thus a repeat ECG-gated study may be redundant among some individuals [33].

Our analysis confirms that the presence of coronary artery calcium on routine chest CT scans predicts the risk of MACE, including transient ischemic attack, stroke, ST-elevation myocardial infarction, and non-ST-elevation myocardial infarction. Indeed, over one-third of the patients included in the study went on to experience a MACE. The most common MACE was stroke, which is consistent with prior studies that have established coronary artery calcium as an independent stroke predictor [34]. There was no significant association between age and MACE. However, patients with coronary artery calcium and comorbid conditions, including diabetes mellitus, heart failure, and hypertension, faced a significantly increased risk of MACE as compared to individuals without known comorbidities.

The association between incidentally detected coronary artery calcium and the risk of MACE has been described by other authors. In a 2021 retrospective cohort study by Yu et al., investigators reviewed the chest CT scan findings of patients without a known history of coronary artery disease and found that the presence of visible coronary artery calcium was independently predictive of MACE [35]. Shao et al. described similar findings in a retrospective single-center cohort study: visually detected coronary artery calcium was a strong independent predictor of both non-fatal myocardial infarction and all-cause mortality [36]. Our findings are consistent with those described by the Yu and Shao groups and provide further evidence that careful evaluation of the coronary arteries on routine chest CT scans provides important clinical information and may allow for early risk modification. 

There are several challenges that must be addressed to improve reporting of coronary artery calcium and ensure timely risk stratification. First, coronary artery calcium goes unrecognized or unreported in nearly half of patients undergoing a CT scan of the chest for other clinical indications. In one analysis of chest CT scans performed in the emergency setting, coronary artery calcium was described in only 55% of patients with positive findings on retrospective review [13]. Furthermore, in cases in which coronary artery calcium is identified, the presence of coronary artery calcium is rarely included within the impression of the report [37]. Second, a standardized reporting mechanism to describe the extent of coronary artery calcium remains to be widely implemented within the radiology community. The SCCT published the Coronary Artery Calcium Data and Reporting System (CAC-DRS) in 2018, which introduced a visual scoring classification schema that can be used to describe coronary artery calcium on routine, non-contrast-enhanced chest CT scans. In CAC-DRS, coronary artery disease is described as absent, mild, moderate, or severe based on the extent and distribution of coronary artery calcium and the presence or absence of extra-coronary calcification. Patients with mild coronary artery calcium by visual assessment are considered candidates for a moderate-intensity statin, whereas those with severe coronary artery calcium should be treated with a high-intensity statin with low-dose aspirin [38]. The CAC-DRS system has been validated in several subsequent studies and has been shown to significantly improve risk stratification [39,40]. However, CAC-DRS is seldom used in clinical practice and is somewhat limited by a lack of standard reference criteria from large-scale clinical studies [41].

There are several limitations to our study inherent to its design. Our analysis was conducted at a single center, and thus events occurring outside of our healthcare system could not be included, which could represent ascertainment bias. We were unable to determine when pharmaceutical agents were initially prescribed. In addition, data pertaining to the specialty of the providers ordering CT scans was not available for review, and therefore selection bias cannot be entirely excluded. Coronary artery calcification assessment was qualitative, and therefore severity data was not available for analysis. We are therefore unable to determine if, for example, lipid-lowering therapies were started before or after a MACE. Despite the use of regression adjustment to account for the likely non-random assignment of lipid-lowering medications, the observational nature of this study limits our assessment of the effect of hyperlipidemia treatment on the occurrence of the composite outcome. Therefore, a randomized controlled trial is needed to assess the effect of lipid-lowering drugs on the occurrence of MACE and cardiovascular death in patients with incidental coronary artery calcium.

Despite these limitations, our analysis provides important data on the relationship between coronary artery calcium and MACE risk and confirms the clinical utility of communicating coronary artery disease on routine chest CT scans. The median age of the subjects in our study was 72 years, which represents an older patient population compared to previous studies. Our findings may therefore be applied to elderly patients who are incidentally found to have CAC on routine chest CT scans. Although we anticipate that the accumulation of comorbid conditions may be an independent risk factor for MACE within this population, we controlled for these factors and age as covariates in our multivariate analysis. Furthermore, the time-to-event analysis excluded subjects with a prior MACE and accounts for the potential for time-varying occurrence of these potential risk factors. In those with incidental CAC on a non-gated chest CT scan, the residual risk for MACE remains high despite lipid-lowering therapies and antiplatelet agents, and close attention is needed to ensure that those therapies are optimized. Future clinical trials should aim to assess the association between cardiovascular mortality and distribution of coronary artery calcium, evaluate the relationship between coronary artery calcium and competing mortality risks, and establish a standardized reporting system that can be widely implemented by radiologists and easily interpreted by referring providers.

## Conclusions

The incidental detection of CAC on routine chest computed tomography scans has important implications for cardiovascular disease risk stratification. In this cross-sectional analysis, patients with CAC were found to be at high risk for MACE. Notably, the residual risk for MACE remained high despite lipid-lowering therapy and antiplatelet agents. The findings suggest that the presence of CAC on routine chest CT scans should prompt early and aggressive lifestyle modification and pharmacologic therapy. In addition, given the close association between CAC and MACE, cardiologists and other providers who play a central role in promoting lifestyle modification and risk mitigation should consider acquiring dedicated coronary artery imaging in the appropriate clinical setting.
